# *In vitro* flow cytometry-based screening platform for cellulase engineering

**DOI:** 10.1038/srep26128

**Published:** 2016-05-17

**Authors:** Georgette Körfer, Christian Pitzler, Ljubica Vojcic, Ronny Martinez, Ulrich Schwaneberg

**Affiliations:** 1 RWTH Aachen University, Worringerweg 3, D-52074 Aachen, Germany; 2 DWI an der RWTH Aachen e.V, Forckenbeckstraße 50, 52056 Aachen, Germany

## Abstract

Ultrahigh throughput screening (uHTS) plays an essential role in directed evolution for tailoring biocatalysts for industrial applications. Flow cytometry-based uHTS provides an efficient coverage of the generated protein sequence space by analysis of up to 10^7^ events per hour. Cell-free enzyme production overcomes the challenge of diversity loss during the transformation of mutant libraries into expression hosts, enables directed evolution of toxic enzymes, and holds the promise to efficiently design enzymes of human or animal origin. The developed uHTS cell-free compartmentalization platform (InVitroFlow) is the first report in which a flow cytometry-based screened system has been combined with compartmentalized cell-free expression for directed cellulase enzyme evolution. InVitroFlow was validated by screening of a random cellulase mutant library employing a novel screening system (based on the substrate fluorescein-di-β-D-cellobioside), and yielded significantly improved cellulase variants (*e.g.* CelA2-H288F-M1 (N273D/H288F/N468S) with 13.3-fold increased specific activity (220.60 U/mg) compared to CelA2 wildtype: 16.57 U/mg).

Throughput in screening is the key criterion for successful directed enzyme evolution and discovery of novel enzymes. Medium to high throughput screening systems based on microtiter plates (MTP) or agar plate formats usually offer to sample 10^4^–10^5^ variants, which is orders of magnitude lower compared to the size of random mutant libraries (10^8^–10^9^)^1^,^2^. Ultrahigh throughput enzyme screening (uHTS) technologies enable a throughput of up to 10^7^ events per hour and are a key tool for today’s state-of-the-art directed evolution experiments^3^,^4^,^5^. Ultrahigh throughput of 10^7^ events per hour enables an efficient coverage of the generated sequence space and exploration of novel directed evolution strategies with high mutational loads^6^,^7^,^8^,^9^. Therefore throughput is decisive to explore the generable diversity and to enable efficient re-engineering of biocatalysts (*e.g.* for chemical, pharmaceutical, food industry) in a time and cost efficient manner^10^.

Employing flow cytometry screening systems with whole cells has been reported in several studies, which have been excellently reviewed^11^,^12^,^13^,^14^,^15^. Recently, a conceptionally novel and robust principle named Fur-Shell was reported for a phytase^16^, a cellulase, a lipase and an esterase^17^ in which fluorogenic microgels have been generated around *E. coli* cells expressing active hydrolase variants. All whole cell-based screening systems have in common that selection is performed on single cells based on a fluorescence signal. Analyzed cells are in varied metabolic states and varied induction times so that a subsequent screen is usually required (*e.g.* in MTP) to determine best performing variants.

The concept of *in vitro* compartmentalization was firstly introduced by Tawfik and Griffiths showing successful cell-free production of dihydrofolate reductase and *Hae*III methyltransferase within (w/o) single emulsion compartments^18^. A significant technological advancement was coupling of flow cytometer-based ultrahigh throughput screening with *in vitro* enzyme library production within water-in-oil-in-water (w/o/w) emulsion compartments (InVitroFlow)^19^. In total three reports have been published which employ InVitroFlow for directed enzyme evolution^20^,^21^,^22^. Reports comprise either screening system validation on a model enzyme library (*i.e.* [FeFe] hydrogenase)^22^, usage of a microbead antibody-antigen-based display in order to maintain the genotype-phenotype linkage^21^, and directed β-galactosidase evolution which resulted in a significantly improved enzyme variant after two rounds of sorting (0.0315 k_cat_/K_M_ to 10.0 k_cat_/K_M_; 317-fold improvement)^20^.

Water-in-oil (w/o) single emulsions are generated using extrusion or homogenizing and contain an inner aqueous phase containing a gene mutant library, a cell-free transcription-translation reaction mixture, and a fluorogenic detection system for activity^19^. The inert oil phase of emulsion compartments mimics the bacterial cell membrane by encapsulating ideally one DNA molecule per compartment and thereby enabling genotype-phenotype linkage^23^. Upon *in vitro* transcription-translation of the mutant library into enzyme variants, active variants convert a fluorogenic substrate into a fluorescent product thus labelling water-in-oil (w/o) emulsions. In order to enable subsequent analysis and sorting by flow cytometer in an aqueous environment, the (w/o) single emulsions are dispersed in an external water phase resulting in water-in-oil-in-water (w/o/w) emulsions^11^,^19^,^24^. *In vitro* compartmentalization (IVC) technology enables the miniaturization of reaction volumes by production of 10^10^ reaction compartments per milliliter of reaction with diameters ranging from 0.5–10 μm, thus resulting in a dramatic reduction of consumable costs, workload and assay time^19^,^20^,^24^. Several challenging criteria must be matched for a successful discrimination between enzyme variants in InVitroFlow. Mainly, diffusion of fluorogenic compounds or cell-free reaction components from (w/o/w) emulsion compartments has be prevented and stable as wells as monodisperse emulsion compartments have to be formed. Main advantage of *in vitro* compartmentalization in (w/o/w) emulsions is its complete independency from host cells, enabling the production of toxic and membrane bound enzymes^25^,^26^. Additionally, InVitroFlow overcomes the challenge of diversity loss in directed evolution experiments due to low transformation efficiency of expression hosts such as industrially important production organisms as *Bacillus strains*^27^,^28^. The key challenge of the (w/o/w) emulsion technology is its compatibility with *in vitro* protein production. Especially, the hydrophobic oil phase can have an inhibitory effect on the transcription-translation process resulting in an insufficient enzyme production^29^. Additionally, produced enzymes can be inhibited or inactivated at the (w/o) interface or by components of (w/o/w) emulsions (*i.e*. surfactants)^29^.

In this manuscript, an uHTS cell-free compartmentalization platform (InVitroFlow) was developed and validated for *in vitro* directed cellulase (CelA2-H288F) evolution employing fluorescein-di-β-D-cellobioside (FDC) as substrate within (w/o/w) emulsion compartments. The development of InVitroFlow comprised optimization of (w/o/w) emulsion generation, cell-free expression of cellulases within emulsion compartments, and defining of a flow cytometer-based sorting strategy using libraries with defined active-to-inactive ratios. The validation of the developed InVitroFlow platform was performed by identification of an improved cellulase variant upon flow cytometry screening of a random mutant cellulase library.

## Results

The result section is divided into three parts in order to develop and to validate the InVitroFlow technology platform for directed cellulose evolution. In the first section, the principle of InVitroFlow is described; followed by the second section comprising optimization steps using the CelA2-H288F cellulase variant as reference for cell-free production in (w/o/w) emulsions, flow cytometry-based analysis, and sorting. In the third section, InVitroFlow was validated in a directed evolution campaign by flow cytometry-based screening of a random mutagenesis library (epPCR library) with the CelA2-H288F cellulase variant as parent (encoded by a linear template). Sorted variants were expressed in 96-well MTP format and the most beneficial cellulase variant M1 (N273D/H288F/N468S) was partially purified and kinetically characterized.

### Principle of high throughput flow cytometry-based *in vitro* compartmentalization screening platform

**Figure 1:**
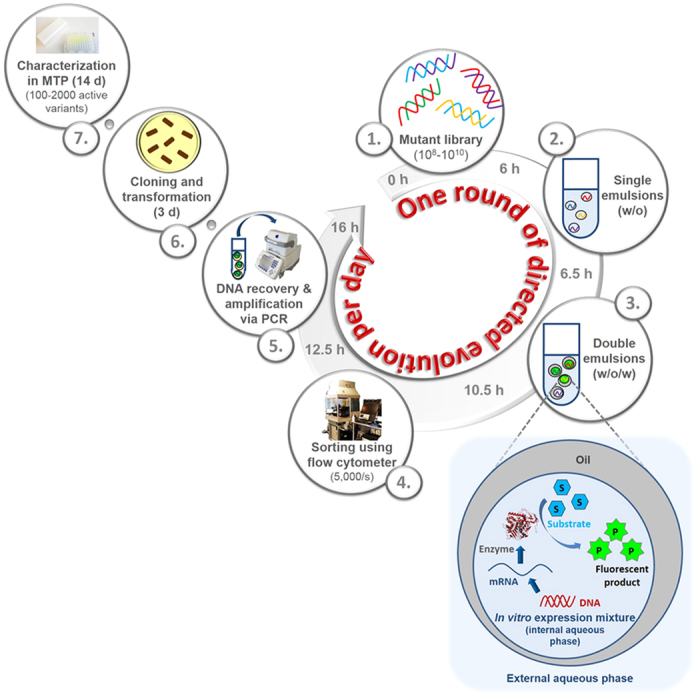
Principle of InVitroFlow comprising 7 steps. (1) Mutant library generation using a linear DNA template (approx. 6 h), (2) entrapment of mutant cellulase library in (w/o) single emulsions within 0.5 h, (3) cell-free expression of mutant library and generation of (w/o/w) emulsions within 4 h, (4) sorting of active variants within (w/o/w) emulsions using flow cytometer within 2 h, and (5) DNA recovery from (w/o/w) emulsions and PCR gene amplification in 3.5 h. A whole round of InVitroFlow (diversity generation, screening by flow cytometry, amplification) can be completed within 16 h. (6) Cloning and transformation into expression host (2 days), and (7) screening of up to 2,000 beneficial clones in MTP format and characterization of a few variants (7–12 days).

Figure 1 summarizes the seven steps of an InVitroFlow-based directed evolution campaign. A *celA2-H288F* library with a high mutational load (8 mutations per gene) containing >10^8^ mutants was generated using epPCR (Fig. 1, Step 1). Subsequently the random mutant library was encapsulated in (w/o/w) emulsion droplets together with supplemented fluorogenic substrate (fluorescein-di-β-D-cellobioside) and an optimized *in vitro* extract mixture for cell-free enzyme production (Fig. 1, Steps 2–3). Optimization comprised selection of optimal substrate and substrate concentration, amount of template DNA, BSA concentration, incubation time and incubation temperature. The compartmentalization in emulsion droplets enables genotype-phenotype linkage since it ensures that the mutated genes, the encoded enzyme variants, and the generated fluorescent products (fluorescein) remain entrapped in the emulsion compartment (Fig. 1, Step 3). As a result, the fluorescent (w/o/w) emulsions contain active enzyme variants which generate sufficient fluorescein for sorting by flow cytometry. InVitroFlow offers a throughput of 10^7^ events per hour and sorting of 5,000 events per second by qualitative differentiation between fluorescent and non-fluorescent events (Fig. 1, Step 4). The genes encoding for active cellulase variants in the sorted sample were isolated and amplified by PCR in approximately 3 hours (Fig. 1, Step 5). Amplified PCR products can subsequently be used as template for further iterative rounds of directed evolution and/or cloned into a vector with a subsequent transformation into an expression host (*e.g.* for MTP screening; Fig. 1, Step 6). MTP-based screening is usually used to identify the most beneficial variants (*e.g*. for improved activity, thermal and organic solvent resistance; Fig. 1, Step 7). In summary, InVitroFlow offers screening of a high number of enzyme variants (>10^8^) within a few hours.

### Optimization of (w/o/w) emulsion compartments

**Figure 2:**
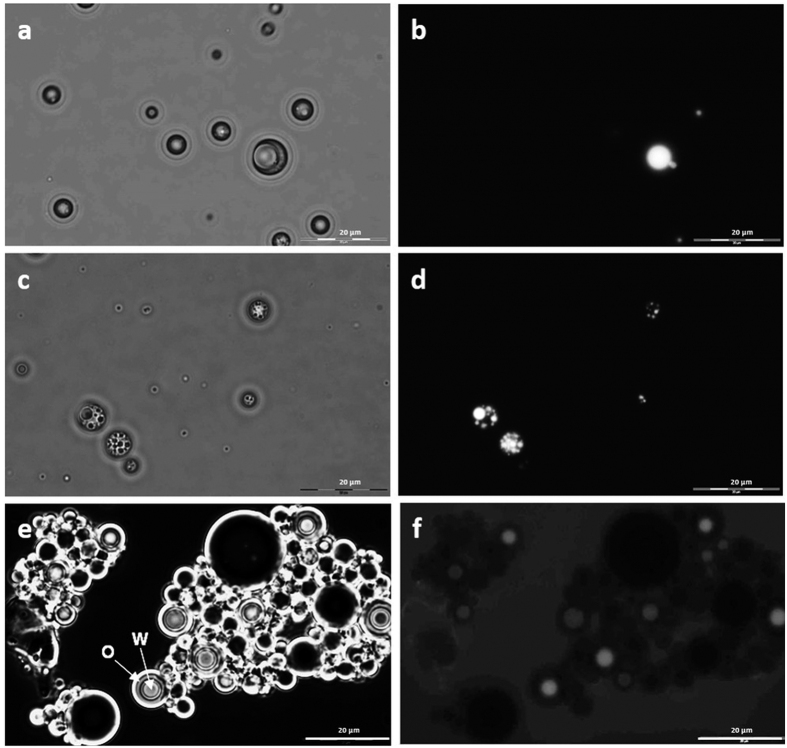
Comparison of methods for (w/o/w) emulsions generation shown as fluorescence microscopic pictures (100x magnification) in light field (a,c,e) and fluorescence field (b,d,f). (**a**,**b**) Stirring method, (**c**,**d**) homogenizing by the Miccra D-1 device (ART Prozess- & Labortechnik GmbH & Co. KG), (**e**,**f**) membrane extrusion method. **W** indicates the inner aqueous phase of the emulsion with an average diameter of 5 μm; **O** indicates the oil phase with an average diameter of 10 μm (**e**). The scale bar represents a length of 20 μm. Sorting parameters of flow cytometry were selected in all subsequent experiments that only single (w/o/w) emulsions with a high fluorescence signal were sorted as beneficial event (see Material and Methods “Flow cytometry-based screening and sorting”).

Essential for high level *in vitro* protein production in (w/o/w) emulsions are: (1) compatibility of cell-free expression mixture and substrate/product with emulsion building components (*i.e.* oil, surfactants), (2) stability throughout the selection procedure (including sorting), (3) homogeneity in size and shape, (4) one inner aqueous phase per (w/o/w) emulsion droplet. Generation of (w/o/w) emulsions was performed by three preparation methods based on stirring, homogenizing and membrane extrusion. The generated emulsions were characterized according to the above mentioned criteria (1–4). Stirring resulted in a comparably low number of (w/o/w) emulsions and a broad size distribution (1–30 μm; Fig. 2a,b). (W/o/w) emulsions obtained using the Miccra D-1 homogenizer (ART Prozess- & Labortechnik GmbH & Co. KG) resulted in polydisperse (w/o/w) emulsions of various sizes (1–20 μm) harboring more than one inner aqueous compartment per droplet (Fig. 2c,d). Membrane extrusion was finally used for InVitroFlow since it yielded highly monodisperse (w/o/w) emulsions of homogeneous size (~10 μm) that harbor only one inner aqueous compartment per droplet (Fig. 2e,f). Fluorescence microscopy analysis over time showed that generated (w/o/w) emulsions have a stability of at least 24 hours under the selected conditions.

### Substrate selection for the InVitroFlow screening platform

In order to maintain genotype-phenotype linkage between gene and encoded cellulase a suitable substrate-product pair was identified. 4-Methylumbelliferyl-β-D-cellobioside (4-MUC) and fluorescein-di-β-D-cellobioside (FDC) were finally used to visualize cellulase catalyzed conversion and leakage of fluorescent product from (w/o/w) emulsions. The pair FDC/fluorescein is more sensitive than the commonly employed coumarin-based substrates^30^ and had lower product diffusion rates from the inner aqueous phase (Fig. 2f). The presence of the charged carboxyl group in FDC probably reduces product leakage into the hydrophobic oil phase and makes FDC a suitable substrate for flow cytometry-based screening in (w/o/w) emulsions. In an additional optimization step, BSA (1–2%) was supplemented to the (w/o/w) emulsions to minimize interfacial interactions and leakage of FDC/fluorescein from (w/o/w) emulsions. The optimal BSA concentration was determined by flow cytometer analysis to be 1 mg/ml BSA (1% BSA, 26.45% of fluorescent events; Supplementary Fig. S1b). BSA-influence on fluorescein leakage from (w/o/w) emulsion compartments was quantified by comparison to samples without BSA (4.75% fluorescent events, Supplementary Fig. S1a) and samples containing 2 mg/ml BSA (2% BSA, 2.50% fluorescent events, Supplementary Fig. S1c).

### Cell-free cellulase production within (w/o/w) emulsion compartments

Cell-free cellulase production in (w/o/w) emulsion compartments generated by the membrane extrusion method was performed using an *in vitro* expression kit (FastLane *E. coli* Mini Kit; RiNA GmbH). Incubation time in the cell-free production mix in (w/o/w) emulsions was varied from 3 h to 24 h and after 4 h a sufficient cellulase activity for flow cytometry analysis (40.3% active fraction) was obtained (Supplementary Fig. S2b). Incubation at varied temperatures revealed an optimal level of cellulase activity at 25 °C which was subsequently used as temperature of choice (Supplementary Figs S2 and S3).

In order to define an efficient sorting window for libraries with a high mutational load, mutant libraries with a defined active-to-inactive ratio (*e.g.* 3:7 ratio of CelA2-H288F_active_ versus CelA2-H288F-E580Q_inactive_) were used to mimic conditions under which mutant libraries are often screened. A model library with an active-to-inactive cellulase ratio of 3:7 was finally used to optimize the amount of template DNA (0.164–0.656 μM) and substrate concentration (0.13–0.75 mM of FDC) for flow cytometer analysis. Samples containing <0.656 μM of template DNA during *in vitro* cellulase production in (w/o/w) emulsions yielded upon flow cytometry analysis fluorescence intensities comparable to the negative control (Supplementary Fig. S4a). A DNA template concentration of 0.656 μM showed 23.9% of fluorescent events upon flow cytometry analysis which is sufficient to efficiently sort mutant libraries containing a fraction of active clones <30% (Supplementary Fig. S4b). Varying substrate concentrations (0.13–0.75 mM FDC) revealed a favorable fluorescence signal-to-noise ratio (17.7-fold increased fluorescence) at 0.46 mM FDC (Supplementary Fig. S5a,b). Higher substrate concentrations (0.75 mM FDC) resulted in no further increase of the fluorescent signal compared to 0.46 mM FDC. At a concentration of 0.75 mM of FDC the signal-to-noise ratio was reduced (9.1-fold) likely due to the higher background signal of the fluorogenic substrate (Supplementary Fig. S5). Finally, the optimal conditions (0.6561 μM DNA, 0.46 mM FDC, 1% BSA and incubation at 25 °C for 4 h) were used for analysis and sorting of the cellulase mutant library within InVitroFlow experiments.

### Flow cytometry analysis and sorting of *in vitro* cellulase DNA model libraries with defined active-to-inactive ratio

**Figure 3:**
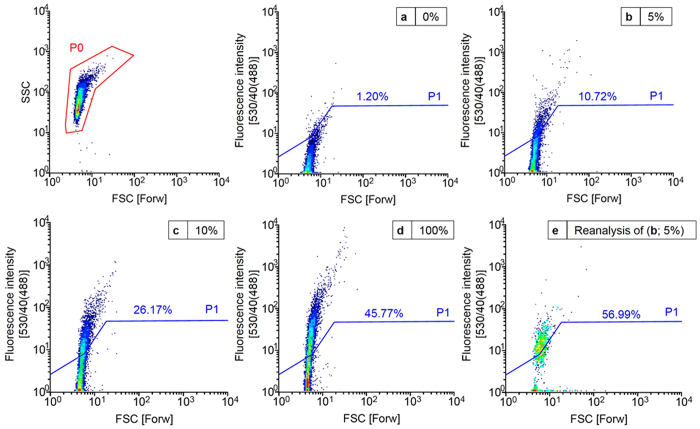
Flow cytometer analysis showing density plots of cellulase model libraries with defined ratios of active and inactive DNA template after *in vitro* expression in (w/o/w) emulsions. The P0 gated area represents the (w/o/w) emulsion population using the FSC representing the size of the (w/o/w) emulsion and the SSC representing the granularity of the (w/o/w) emulsion. Y-axis represents the fluorescence intensity after 488 nm excitation with a fluorescence emission detection between 530 ± 20 nm which is annotated by 530/40(488). Lines indicate gate P1 which was set to categorize events in highly fluorescent and potentially beneficial events (appearing above) and not beneficial events. (**a**) Negative control with CelA2-H288F-H580Q_inactive_ DNA: Forward scatter (FSC) versus side scatter (SSC) and FSC versus fluorescein fluorescence intensity (λ_ex_ 494 nm and λ_em_ 516 nm), (**b**) 5% model library (ratio 5:95 of CelA2-H288F_active_ versus CelA2-H288F-E580Q_inactive_), (**c**) 10% model library (ratio 10:90 of CelA2-H288F_active_ versus CelA2-H288F-E580Q_inactive_), (**d**) Positive control with CelA2-H288F_active_, and (**e**) Reanalysis of the sorted 5% model library (**b**) resulted in 5.3-fold enrichment of the active events above the line P1.

Cell-free expressed DNA model libraries with an active-to-inactive ratio of 5:95 and 10:90 (CelA2-H288F_active_ versus CelA2-H288F-E580Q_inactive_) in (w/o/w) emulsions were analyzed for determining parameters for efficient flow cytometry sorting. Analysis and sorting was done at an event rate of 1,500 events s^−1^. Figure 3 shows that an increase in the amount of CelA2-H288F_active_ leads to an increase in the fluorescent signal above the line indicating gate P1. In case of 0% CelA2-H288F_active_, 1.2% of the fluorescent population is located above the line P1 that was used for sorting active variants (Fig. 3a). Analysis of 5% of a CelA2-H288F_active_ population results in approx. 11% positive events above the line P1 (Fig. 3b), whereas for 10% CelA2-H288F_active_, 26% of the population was above the line P1 (Fig. 3c). Analysis of a 100% CelA2-H288F_active_ population shows that 46% of the population is above the line P1, (Fig. 3d). A 46% fraction of active events in the positive control (100% CelA2-H288F_active_) suggests that not all (w/o/w) emulsions contain a gene template. The latter is due to the applied dilution conditions by emulsification of the gene sample which are randomly loaded according to Poisson statistics into (w/o/w) emulsions^31^. In order to ensure that the majority of emulsions contains only one gene per droplet the majority of emulsion compartments has to be without any gene. The library with an active-to-inactive ratio of 5 to 95 was sorted at an event rate of 1,500 events s^−1^ with a sorting efficiency of 99.6%. Reanalysis of the sorted fraction resulted in a 5.3-fold enrichment of the active emulsion fraction (Fig. 3e). The obtained enrichment validated the InVitroFlow technology platform since it is possible to isolate a significantly increased number of fluorescent events (∼57%).

### Flow cytometry screening and sorting epPCR libraries

**Figure 4:**
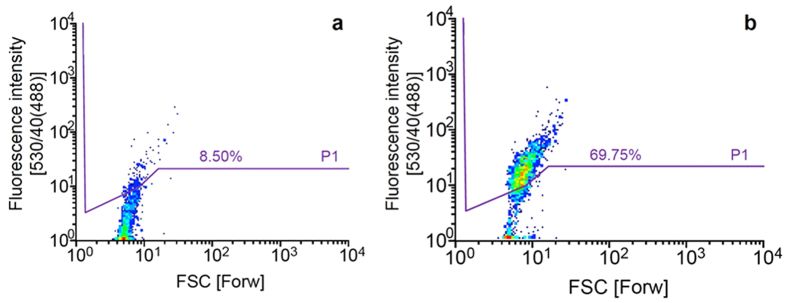
Flow cytometer analysis showing density plots of random *celA2-H288F* mutant library after *in vitro* expression in (w/o/w) emulsions (4 h, 25 °C). The Forward scatter (FSC) represents the size of the (w/o/w) emulsion and the y-axis represents the fluorescein fluorescence intensity (λ_ex_ 494 nm and λ_em_ 516 nm) after 488 nm excitation with a fluorescence emission detection between 530 ± 20 nm which is annotated by 530/40(488). Lines indicate gate P1 separating fluorescent events (appearing above) from non-fluorescent events. (**a**) *celA2-H288F* random mutant library during sorting of the 8.5% most fluorescent events. (**b**) Reanalysis of sorted library with an 8.2-fold enriched active fraction (69.75% fluorescent events).

A random mutant library of *celA2-H288F* was generated by epPCR using 0.05 mM MnCl_2._ The random mutant library of *celA2-H288F* was subsequently employed in the InVitroFlow screening system with the previously optimized conditions. An average mutation frequency of 8 mutations per gene was estimated by sequence analysis of 12 randomly selected clones. In order to achieve high enrichment in the active fraction (>50%) a strict sorting strategy was applied to sort out the single (w/o/w) emulsion events with the highest fluorescence (8.5% fluorescent events above the line P1) (Fig. 4a,b). Upon flow cytometry analysis of the cell-free expressed *celA2-H288F* random mutant library in (w/o/w) emulsions in total 1.4 × 10^7^ events were analyzed (2,000 events s^−1^), and the 8.5% most fluorescent population (above the line P1) was sorted with a 93.2% efficiency (Fig. 4a). Reanalysis of the sorted population revealed an enrichment in active fraction of 8.2-fold, determined by an increase of the population above the line P1 from 8.50% to 69.75% (Fig. 4b).

### Recovery of sorted DNA library from (w/o/w) emulsions and transformation in expression host for MTP analysis

**Figure 5:**
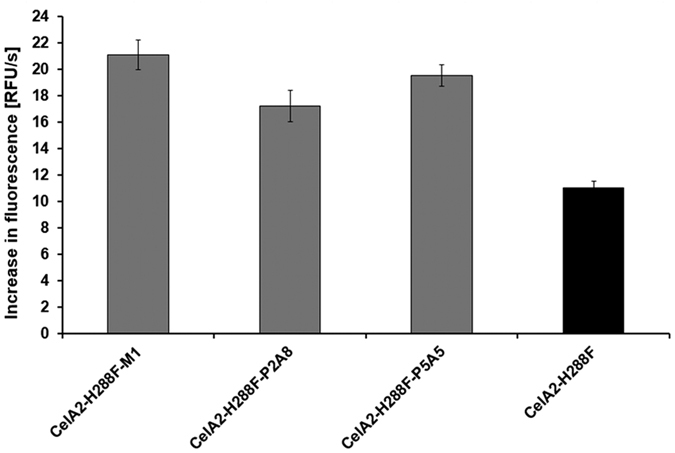
Results of directed evolution of CelA2-H288F by an InVitroFlow campaign with a single round of random mutagenesis and flow cytometer-based screening. Rescreening in MTP using 4-MUC assay of the three best variants in comparison to parent CelA2-H288F (black); the x-axis describes the different variants and the y-axis shows the increase in fluorescence in relative fluorescent units (RFU) per s. The reported values are the average of ten measurements and the shown average deviations are calculated from the mean values.

The sorted (w/o/w) emulsions showing highest fluorescence were disrupted and an optimized DNA recovery method based on NucleoSpin^®^ Gel and PCR clean up kit (Macherey-Nagel) was used to isolate the DNA of the mutant library. After PCR amplification the PCR products of *celA2* were analyzed using agarose-gelelectrophoresis (Supplementary Fig. S6), cloned into the pET28a(+) vector backbone *via* PLICing^32^, and transformed into *E. coli* BL21 Gold (DE3) for expression. In total, 528 cellulase variants were screened with the coumarin based 4-MUC activity quantification system in 96-well MTPs^30^. MTP analysis revealed 33 cellulase variants with significantly improved activity in comparison to the parent CelA2-H288F (up to 7.2-fold) even though that 4-MUC and not the pair FDC/fluorescein was used in the rescreening. The latter indicates that a high quality library was isolated in which also activities for other substrates than the screening substrate can be optimized. Analysis of variant CelA2-H288F-M1 showed 17.5-fold improvement for the pair FDC/fluorescein in comparison to the parent CelA2-H288F (Supplementary Fig. S7). The three most promising variants were rescreened and finally CelA2-H288F-M1 (N273D/N468S) revealed to be the most beneficial cellulase variant (Fig. 5).

### Purification and kinetic characterization of CelA2 and its variants

CelA2 wild type, CelA2-H288F parent, and variant CelA2-H288F-M1 (N273D/N468S) were purified (>86% of the total protein content) and the kinetic parameters (k_cat_, K_M_) were determined with the 4-MUC detection system (Table 1). Identified variant CelA2-H288F-M1 (N273D/N468S) showed a comparable K_M_ value to the CelA2-H288F parent. The determined specific activity value of CelA2-H288F-M1 (N273D/N468S) is 13.3-fold higher compared to CelA2 wild type (gain 204.03 U mg^−1^) and 3.0-fold higher (gain 147.98 U mg^−1^) compared to its CelA2-H288F parent (Table 1). The calculated specific activity of CelA2-WT is in agreement with the published data from Lehmann *et al*. (15.1 U mg^−1^)^30^.

## Discussion

Protein engineering by directed evolution is limited by the complexity of the protein sequence space. Already a peptide with five amino acids can yield 3.2 million different proteins. In traditional directed evolution campaigns only a minor fraction of clones are screened and often already screening of a few thousands of clones yields improved variants^33^. In a comprehensive study the groups of Jaeger and Schwaneberg showed that 70 to 80% of beneficial positions are discovered in traditional directed evolution campaigns^34^,^35^. Therefore, a higher throughput with a balanced mutations frequency is of very high importance to efficiently capitalize on nature’s diversity.

The InVitroFlow screening platform combines cell-free compartmentalization with flow cytometer-based analysis and achieves a throughput of 6.5 × 10^6^ events per hour. In a single round of directed cellulase evolution over 1.4 × 10^7^ events were screened. Rescreening in MTP format revealed the highly improved cellulase variant M1 (N273D/H288F/N468S; specific activity of 220.6 U mg^−1^) compared to CelA2 wild type (16.57 U mg^−1^), and the parent CelA2-H288F (72.62 U mg^−1^) despite that a coumarin derivative (4-MUC) and not flow cytometer prescreening substrate (pair FDC/fluorescein) was employed in the MTP rescreen. Cellulase InVitroFlow represents the first *in vitro* compartmentalization-based flow cytometry screening system for directed cellulase evolution and it is the first report in the last ten years. In total up to now, only three directed enzyme evolution campaigns with InVitroFlow were reported^20^,^21^,^22^. InVitroFlow technology enables to sample up to 10^10^ events^21^,^36^ which represents well coverage of the generated diversity number of a random mutant library (>10^6^)^37^. InVitroFlow enables to sample variant numbers that are order of magnitude higher than standard whole cell screening systems (>10^3^-fold)^36^,^38^ despite that InVitroFlow requires oversampling; only every 100^th^ emulsion droplet contains a gene template to ensure that sorted emulsions contain genes which encode in the majority beneficial variants so that diversity loss in subsequent PCR amplification is minimized.

Challenges for the rare usage of *in vitro* emulsion compartmentalization combined with flow cytometry lie in the limited efficiency of *in vitro* enzyme production within water-in-oil-in-water emulsion compartments and the confinement of the fluorescence signal in emulsions. The hydrophobic oil phase in emulsions mimicking the cell membrane causes interfacial inactivation of the produced enzyme^29^ and expression kit components. In the cellulase InVitroFlow screening system, the cellulase production within (w/o/w) emulsions was optimized using the BSA protein as “sacrificial” substrate for “saturation” of the water/oil interface which yielded a 5-fold increase in fluorescence signal. Additionally, the employed fluorogenic substrate was directly encapsulated in (w/o/w) emulsion compartments and the presence of the negatively charged carboxylic group minimizes leakage and crosstalk.

The application potential and industrial opportunities of *in vitro* evolution of enzymes, especially enzymes from human or animal origin beyond standard bacterial and yeast proteins, is very impressive as summarized in several reviews^39^,^40^. Expression of eukaryotic proteins is mainly performed in wheat germ extracts (production of up to mg/ml amounts of proteins), while cell-free extracts of insect and *E.coli* cells are characterized by lower protein yields and production of nascent or insoluble proteins, respectively^41^. Cell-free expression offers the opportunity to express toxic^25^,^42^,^43^ and membrane proteins^44^,^45^ for which *in vivo* expression remains challenging. In the last five years, successful expression of three toxic proteins *i.e.* perisin-like protein from the cabbage butterfly *Pieris rapae*^43^, hemolysins of *Vibrio parahaemolyticus*^42^*, and* expression with the toxic amino acid canavanine^25^ was reported. Most commonly used production strains in industry *i.e. Aspergillus niger, Bacillus* and *Trichoderma* are often limited in transformation efficiencies^28^,^46^ and directed evolution in *E. coli* or yeast strains can yield different results due to differences in glycosylation or result in further codon usage optimization for high level expression. Recent reports on usage of novel eukaryotic and vesicle containing cell-extracts, *i.e.* insect extracts^41^ and human-based cell-free extracts^47^ enabled the N-linked glycosylation of the target protein and the embedding of membrane proteins into microsomal membranes, which is of great interest for the scientific and industrial community^40^,^48^.

In summary, Cellulase InVitroFlow is a rapid, cost-efficient and non-laborious screening platform for directed cellulase evolution which offers to cover a significant fraction of the generated sequence space and to identify beneficial positions beyond the possibility of traditional screening formats. We see the main application of the InVitroFlow technology as prescreening system enabling researchers to isolate the most active variants from a vast pool of variants and to explore novel directed evolution strategies with high mutational loads in which only a small fraction of a populations is active. Advancing InVitroFlow to cell free expression systems based on wheat germ and other eukaryotic cell extract will be challenging and will open many exciting possibilities to evolve with an InVitroFlow prescreen cell toxic proteins and proteins from human or animal origin.

## Materials and Methods

### Materials

All chemicals used were of analytical reagent grade or higher quality and purchased from Sigma-Aldrich Chemie GmbH (Taufkirchen, Germany), Applichem (Darmstadt, Germany), or Carl Roth (Karlsruhe, Germany). All enzymes were purchased from New England Biolabs (Frankfurt, Germany). Fluorogenic substrate Fluorescein di-β-D-cellobioside (FCB) was synthesized by AAT Bioquest, Inc. (Sunnyvale, CA, USA) and purchased from BIOMOL GmbH (Hamburg, Germany). Fluorogenic substrate 4-Methylumbelliferyl-β-D-cellobioside (4-MUC) was purchased from Sigma–Aldrich (Saint–Louis, USA). *In vitro* transcription-translation kit was purchased from RiNA GmbH (Berlin, Germany). Plasmid isolation, PCR purification and His-tag purification kits were purchased from Macherey-Nagel GmbH & Co. KG (Düren, Germany). Microtiter plates (96-wells) for cultivation (Greiner Bio-one GmbH, Frickenhausen, Germany) and *in vivo* protein expression (Corning Incorporated, New York, USA) were incubated in a Multitron II Infors shaker (Infors AG, Bottmingen, Switzerland).

In all PCRs a thermal cycler (Mastercycler gradient; Eppendorf, Hamburg, Germany) and thin-wall PCR tubes (Multi ultra-tubes; 0.2 ml; Carl Roth GmbH, Karlsruhe, Germany) were used. DNA concentrations in all experiments were quantified using a NanoDrop photometer (ND-1000, NanoDrop Technologies, Wilmington, DE, USA). For detection of fluorescence Tecan Infinite^®^M1000 (Tecan Group Ltd., Männedorf, Switzerland) plate reader (λ_ex_: 488 nm, λ_em_: 530 nm for FCB and λ_ex_.: 330 nm, λ_em_: 450 nm for 4-MUC) or the flow cytometer (BD Influx Cell Sorter, Becton Dickinson Biosciences, Erembodegem, Belgium) were used. The Experion system from Bio-Rad (München, Germany) and Pierce^®^ BCA Protein Assay Kit (No. 23225, Life Technologies GmbH, Darmstadt, Germany) were used for estimation of protein concentrations and protein purity analysis according to the manual’s instructions.

### Strains, plasmids, and target gene

*E.coli* DH5α (Stratagene, La Jolla, CA, USA) and *E.coli* XL10 Gold (Stratagene, La Jolla, CA, USA) were used as cloning hosts. *E.coli* BL21 Gold (DE3) (Aglient Technologies, Santa Clara, CA, USA) and *E.coli* BL21 Gold (DE3)-lacIQ^1^^ ^32 were used for the expression and generation of epPCR cellulase libraries. Plasmids pET-28a(+) from Novagen (Darmstadt, Germany) and pIX3.0-RMT7 modified in house using Qiagen vector pIX3.0 (Hilden, Germany), were used as *in vivo* and *in vitro* expression vectors.

The gene *celA2* (GenBank: JF826524.1), isolated from a metagenome library, with 41% similarity to a Glycosyl Hydrolase Family 9 (GH9) cellobiosidase from *Clostridium cellulovorans* codes for a cellulase protein with a molecular weight of ∼69 kDa, consisting of 604 amino acids^49^. Positions 288 and 580 are reported to be key residues for activity, so in this study variant CelA2-H288F, showing 8.7-fold higher specific activity than CelA2-WT, was used for development of the uHTS IVC platform^30^. The amino acid substitution from glutamic acid to glutamine at the catalytic residue 580 is reported to lead to a complete loss of activity, therefore variant CelA2-H288F-E580Q, showing a behavior like the empty vector pET28a(+), was used as negative control in this study^50^.

### Gene cloning into expression vectors and sequencing

The construct pET28a(+)-CelA2-H288F was modified by introducing an additional restriction *Nde*I site in front of His-tag sequence by PCR. Subsequently *celA2-H288F* gene was cut out using double restriction (*Nde*I and *Xho*I) and subcloned into pIX3.0RMT7 vector backbone. The *Nde*I-*celA2-H288F* insert was generated by PCR (98 °C for 30 s, 1 cycle; 98 °C for 15 s/63 °C for 15 s/72 °C for 90 s, 25 cycles; 72 °C for 5 min, 1 cycle) using dNTP mix (0.2 mM), primers (NdeI_CelA2_FW: 5′-atataacatATGGGTAGCAGCCATCAC-3′; T7_pET28_REV: 5′–GTTATTGCTCAGCGGTGGCAGCAGC-3′; 0.4 μM each), plasmid DNA template (pET28a(+)-CelA2-H288F, 20 ng), NEB Phusion HF polymerase (2.5 U) in a final volume of 50 μl. The amplified PCR product was digested (1 h, 37 °C) by *Dpn*I (20 U), purified using the NucleoSpin^®^ Gel and PCR clean up kit (Macherey-Nagel GmbH & Co. KG, Düren, Germany) and eluted in 20 μl ddH_2_O. Ligation was performed according to the manufacturer’s recommendations (New England Biolabs, Frankfurt a. Main, Germany). The recombinant plasmid was named pIX3.0RMT7-CelA2-H288F.

The inactive variant CelA2-H288F-E580Q was generated by site directed mutagenesis according to the published method^51^ using pIX3.0RMT7-CelA2-H288F as template DNA and specific SDM primers (SDM_E580Q_for: 5′-GCTATGCCACCAAT**CAG**ATTTGCATTTATTGGAATAGTCCG-3′, SDM_E580Q_rev: 5′-CGGACTATTCCAATAAATGCAAAT**CTG**ATTGGTGGCATAGC-3′). The recombinant plasmid was named pIX3.0RMT7-CelA2-H288F-E580Q. Constructs were digested (1 h, 37 °C) by *Dpn*I (20 U), purified using NucleoSpin^®^ Gel and PCR clean up kit (Macherey-Nagel) and transformed into chemically competent *E.coli* BL21 Gold (DE3)-lacIQ^1^ cells. DNA sequencing of the inserted gene was performed by Eurofins Genomics (Ebersberg, Germany) and Clone Manager 9 Professional Edition (Sci-Ed software, Cary, USA) was used for sequence analysis.

### Assay for the detection of cellulase (CelA2) activity

*Fluorimetric microtiterplate-based assay*. The 4-MUC activity assay was performed in 384-well MTPs (polystyrene, black, flat bottom; Greiner Bio–one GmbH, Frickenhausen, Germany). Reactions had a final volume of 20 μl. For the assay 10 μl fluorogenic substrate 4-MUC (4-Methylumbelliferyl-β-D-cellobioside; No. M 6018, Sigma–Aldrich, Saint–Louis, USA; 0.1 mM dissolved in potassium phosphate buffer, pH 7.2, 0.2 M) was added to 10 μl of cell supernatant or purified enzyme or *in vitro* expressed sample. Enzyme activity was measured at 30 °C by monitoring the increase in fluorescence (λ_ex_ 330 nm, λ_em_ 450 nm), using Tecan Infinite^®^M1000 (Tecan Group Ltd., Männedorf, Switzerland) plate reader.

### Generation of epPCR mutagenesis library of CelA2

The random mutagenesis library was constructed by standard epPCR method^52^. The epPCR library was generated by PCR (94 °C for 1 min, 1 cycle; 94 °C for 30 s/51 °C for 30 s/72 °C for 90 s, 25 cycles; 72 °C for 5 min, 1 cycle) using dNTP mix (0.2 mM), primers (pIX3.0RMT7_FW: 5′-TGCAAGGCGATTAAGTTGGG-3′; pIX3.0RMT7_REV: 5′-ACCCCAGGCTTTACACTTTATG-3′; 0.4 μM each), plasmid DNA template (CelA2-H288F, 20 ng), *Taq* polymerase (2.5 U), and MnCl_2_ (0.05 mM) in a final volume of 50 μl. The amplified epPCR product was digested (1 h, 37 °C) by *Dpn*I (20 U), purified using the NucleoSpin^®^ Gel and PCR clean up kit (Macherey-Nagel), eluted in 15 μl ddH_2_O and used for *in vitro* expression of the linear template library.

### Detection of cellulase activity in emulsions

Upon *in vitro* transcription and translation of the cellulase DNA within the “*in vitro* expression mixture” active cellulases are able to convert the fluorogenic substrate fluorescein-di-β-D-cellobioside (FDC) into the fluorescent product fluorescein and two units of D-cellobiose by cleavage of the β-1,4-D-glycosodic bonds. The product fluorescein can be detected within the (w/o/w) emulsions by fluorescence measurements (λ_ex_ 494 nm, λ_em_ 516 nm) in MTP- or flow cytometry-based assay formats. Due to the presence of charged COO^-^ group, the fluorescein showed low difussion into the oil phase of the emulsion droplet. Emulsion droplets harbouring active cellulase variants show a green fluorescence due to substrate conversion, making FDC applicable for flow cytomtery screening.

### Compartmentalization in emulsions by stirring and homogenizing

The (water-in-oil [w/o]) single emulsions were generated by stirring (15 min, RT) with IKAMAG REO magnetic stirrer (IKA^®^-Werke GmbH & CO. KG, Staufen, Germany) by adding 200 μl of solution A (2.9% (wt/wt) ABIL^®^ EM 90 (Evonik Industries AG Personal Care, Essen, Germany) in light mineral oil) to 100 μl PBS buffer (pH 7.4, 1.06 mM KH_2_PO_4_, 2.97 mM Na_2_HPO_4_, and 155.17 mM NaCl) containing fluorescein (60 μM).

For generation of (w/o) single emulsions by homogenizing 200 μl of solution A (2.9% (wt/wt) ABIL^®^ EM 90 (Evonik Industries AG Personal Care, Essen, Germany) in light mineral oil) were added to 100 μl PBS buffer (pH 7.4, 1.06 mM KH_2_PO_4_, 2.97 mM Na_2_HPO_4_, and 155.17 mM NaCl) containing fluorescein (60 μM) and homogenized (1 min, 5000 rpm) with Miccra D-1 device (ART Prozess- & Labortechnik GmbH & Co. KG, Müllheim, Germany).

The second emulsification was performed subsequently by addition of 1000 μl of solution B (1.5% (wt/vol) Carboxymethylcellulose sodium salt, 2% (wt/vol) Tween 20 in PBS buffer (pH 7.4, 1.06 mM KH_2_PO_4_, 2.97 mM Na_2_HPO_4_, and 155.17 mM NaCl)) to the (w/o) emulsion generated either by stirring or by homogenizing method and homogenized (3 min, 7500 rpm) with Miccra D-1 device (ART Prozess- & Labortechnik GmbH & Co. KG) to obtain (water-in-oil-in-water [w/o/w]) double emulsions for analysis on flow cytometer.

### Compartmentalization by extrusion and cell-free production of CelA2 from linear and plasmid DNA template in emulsions

Cell-free cellulase production was performed in emulsion compartments using the FastLane *E.coli* Mini Kit (RiNA GmbH, Berlin, Germany) in combination with extrusion method. Optimal cell-free cellulase production in (w/o/w) emulsions was achieved by optimizing parameters such as incubation temperature, incubation time, amount of DNA template, substrate concentration and BSA concentration. For successful *in vitro* transcription and translation, a *celA2* gene library (0.656 μM linear epPCR product generated using 0.05 mM MnCl_2_) was mixed with the *in vitro* expression mixture (35 μl *E.coli* extract, 40 μl Easy Xpress reaction buffer, 0.8 μl Chaperone-Mix (DnaK, DnaJ, GroE), 1.15 μl of BSA (100 mg/ml), 27 μl substrate (2 mM; final conc. 0.46 mM FDC), RNase free water added to 115 μl and incubated within (w/o) emulsion compartments (4 h, 25 °C, in water bath).

The (w/o) single emulsions were generated by extrusion method with an Avanti Mini-Extruder (Avanti, Polar Lipids, Inc., Alabaster, USA) by adding 200 μl of W1 solution (1.25 g Span 80, 0.25 g Tween 80 dissolved in 60 ml light mineral oil) to the 115 μl *in vitro* reaction mixture and pushing the solution tree times though 5 μm Whatman membrane (Whatman Nuclepore Track-Etched Membranes, Sigma-Aldrich Biochemie GmbH, Hamburg, Germany) with the extruder device. The (w/o) emulsion sample containing the *in vitro* reaction mixture was collected in a 0.5 ml Eppendorf tube for incubation (4 h, 25 °C). Subsequently the second emulsification was performed by addition of 700 μl of W2 solution (0.5% Tween 80 in PBS buffer (1.06 mM KH_2_PO_4_, 2.97 mM Na_2_HPO_4_, and 155.17 mM NaCl, pH 7.4)) to the (w/o) emulsion and three times extrusion though 12 μm Whatman membrane (Whatman Nuclepore Track-Etched Membranes, Sigma-Aldrich Biochemie GmbH, Hamburg, Germany) to obtain (w/o/w) double emulsions for analysis on flow cytometer.

### Flow cytometry-based screening and sorting

The (w/o/w) emulsions harboring the cell-free expressed CelA2-linear template epPCR library were diluted 1000-fold in PBS buffer (pH 7.4, 1.06 mM KH_2_PO_4_, 2.97 mM Na_2_HPO_4_, and 155.17 mM NaCl), filtered through 50 μm cell trics (Sysmex Partec GmbH, Görlitz, Germany) and analyzed with BD Influx cell sorter (Becton Dickinson Biosciences, Erembodegem, Belgium). Analysis and sorting was performed according to forward and sideward scatter as well as to the fluorescein-based fluorescence intensity (λex 494 nm, λem 516 nm) after 488 nm excitation with a fluorescence emission detection between 530 ± 20 nm (530/40(488)). The BD Influx flow cytometer was operated with a 100 μm nozzle and PBS (pH 7.4, 1.06 mM KH_2_PO_4_, 2.97 mM Na_2_HPO_4_, and 155.17 mM NaCl) was used as sheath fluid. Fluorescence intensities of (w/o/w) emulsions labelled with the converted product fluorescein were recorded, and sort gates were set to collect (w/o/w) emulsions with high green fluorescence intensity. Out of 1.4 × 10^7^ analyzed events 1.0 × 10^6^ were sorted (Sort mode: 1.0 Drop Pure) using 1,800 events s^−1^ analysis speed allowing a throughput of 6.48 × 10^6^ per hour. Reanalysis of the sorted populations with flow cytometer showed 8-fold enrichment of the active (w/o/w) emulsion population. Sorted (w/o/w) emulsions were collected in PBS and genes were isolated by breakage of the (w/o/w) emulsions and purification of the recovered DNA.

### Recovery of DNA from double (w/o/w) emulsions

Samples after sorting were heated up (5 min, 70 °C) to break the emulsions. DNA was recovered from (w/o/w) emulsion sample by purification of sorted (w/o/w) emulsions in PBS (pH 7.4, 1.06 mM KH_2_PO_4_, 2.97 mM Na_2_HPO_4_, and 155.17 mM NaCl) with the NucleoSpin^®^ Gel and PCR clean up kit (Macherey-Nagel) according to the user’s manual. Samples were eluted from the purification column with 15 μl ddH_2_O. DNA amount was measured by Nano Drop 2000 UV-Vis Spectrophotometer (Thermo Scientific, Wilmington, USA). The obtained DNA amount was used as a template in a subsequent recovery PCR (94 °C for 1 min, 1 cycle; 94 °C for 30 s/60 °C for 30 s/72 °C for 2 min, 35 cycles; 72 °C for 5 min, 1 cycle) was performed, containing *Taq* DNA polymerase (2.5 U), dNTP mix (0.3 mM), forward PLICing primer (0.4 μM) (1_FW_Cel_PLIC: 5′-AGCAGCGGTGAAAATCTGTATTTTCAGG-3′), reverse PLICing primer (0.4 μM) (1_RV_Cel_PLIC: 5′-TTCGGCCACGGTATTCAGGCTATAAACAT-3), recovered DNA from (w/o/w) emulsions (20 ng) in a final volume of 25 μl.

### Cloning of enriched, active CelA2 variants into pET28a(+) expression vector

Amplified epPCR libraries (before and after) sorting were cloned into pET28a(+) vector backbone by PLICing for expression and activity analysis in MTP^32^. For vector backbone amplification, the pET28a(+)-CelA2 DNA template was amplified using standard PCR (98 °C for 30 s, 1 cycle; 98 °C for 30 s/57 °C for 30 s/72 °C for 4 min 30 s, 25 cycles; 72 °C for 5 min, 1 cycle), containing *PfuS* DNA polymerase (2.5 U), dNTP mix (0.2 mM), forward PLICing primer (0.4 μM) (2_FW_Cel_PLIC: 5′-ACCGTGGCCGAATAATAAGAATTCGAGC-3′), reverse PLICing primer (0.4 μM) (2_RV_Cel_PLIC: 5′-TTCACCGCTGCTATGATGATGGTGG-3), pET28a(+)-CelA2 DNA (20 ng) in a final volume of 25 μl.

The amplified PCR products were digested (1 h, 37 °C) by *Dpn*I (20 U), analyzed for correct size by agarose-TAE gel electrophoresis according to the standard protocol^53^, purified from agarose gel using the NucleoSpin^®^ Gel and PCR clean up kit (Macherey-Nagel) and eluted in 20 μl ddH_2_O. Purified PCR products were used for PLICing and resulting hybridization products were transformed into 100 μl chemically competent *E. coli* BL21-Gold (DE3) cells following the standard protocol^54^. Transformants were grown on LB/Kan agar plates for further analysis in 96-well plate.

### Cultivation and expression of CelA2 and its variants in 96-well MTP

Transformants were transferred *via* toothpick into 96-well flat bottom MTPs (Greiner Bio-one GmbH, Frickenhausen, Germany) containing 150 μL Luria broth medium supplemented with Kanamycin (50 μg/ml, LB_Kan_) and cultivated (16 h, 37 °C, 900 rpm, 70% humidity). Plates were stored at −80 °C after addition of 100 μl glycerol (50% w/v) as master plates.

For preculture preparation a 96-well flat bottom MTP (Greiner Bio-one GmbH, Frickenhausen, Germany) containing 150 μL LB_Kan_ was inoculated with 96-well replicator from master plate and incubated (16 h, 37 °C, 900 rpm, 70% humidity).

For main culture preparation a 96-well V-bottom expression MTP (Greiner Bio-one GmbH, Frickenhausen, Germany) containing 150 μL Terrific Broth medium supplemented with Kanamycin (50 μg/ml; TB_Kan_) was inoculated with 10 μl of the preculture. After cultivation (2 h, 37 °C, 900 rpm, 70% humidity) 15 μl IPTG (0.01 mM) per well was added for induction of protein expression, plates were incubated (4 h, 30 °C, 900 rpm, 70% humidity), centrifuged (4000 *g*, 10 min, 4 °C) and pellets were stored at −20 °C.

### Cultivation, expression in flask and purification of CelA2 and its variants

For cultivation Luria broth (LB) supplemented with appropriate antibiotics and a shaking incubator (Multitron II; Infors GmbH, Einsbach, Germany) was used for incubation (20 h, 37 °C, 900 rpm). Ampicillin (100 μg/ml) and Kanamycin (50 μg/ml) were used as antibiotics for growth selection.

For *in vivo* cellulase expression the main expression culture was inoculated with 1% of the preculture and incubated until OD_600_ of 0.6 was reached (37 °C, 250 rpm) in 100 ml Terrific Broth (TB) supplemented with appropriate antibiotics. Cellulase expression was induced by addition of 100 μl IPTG (0.1 M). Cells were harvested after incubation (4 h, 30 °C, 250 rpm) by centrifugation (4000 *g*, 10 min, 4 °C) and pellets were stored at −20 °C.

For cell disruption pellets were resuspended in Tris-HCl buffer (pH 7.4, 50 mM), sonicated 3 × 30 s and lysates were centrifuged (13,000 *g*, 15 min, 4 °C). Supernatants were filtered 0.45 μm before His-Tag purification with Protino^®^ Ni-IDA 2000 Packed Columns (Macherey-Nagel GmbH & Co. KG, Düren, Germany) as described in the manufacturer’s protocol. Purity was determined using Experion^TM^ Automated Electrophoresis System (Bio-Rad Laboratories GmbH, München, Germany) and Pierce^TM^ BCA Protein Assay Kit (Life Technologies GmbH, Darmstadt, Germany).

### Determination of kinetic parameters for CelA2 activity with 4-MUC activity assay

For determination of kinetic parameters of purified CelA2-H288F and its variants 4-MUC activity assay was performed according to the published protocol^30^.

## Additional Information

**How to cite this article**: Körfer, G. *et al. In vitro* flow cytometry-based screening platform for cellulase engineering. *Sci. Rep.* **6**, 26128; doi: 10.1038/srep26128 (2016).

## Supplementary Material

Supplementary Information

## Figures and Tables

**Table 1:** Kinetic characterization of CelA2 wild type, CelA2-H288F parent, and variant CelA2-H288F-M1 with the 4-MUC activity-quantification system.

	**k**_**cat**_**[min**^**−1**^]	**K**_**M**_**[μM]**	**k**_**cat**_**/K**_**M**_**[min**^**−1**^ μ**M**^**−1**^]	**Specific activity [U mg**^**−1**^]	**Amino acid exchanges**
CelA2-WT	0.11 (±0.021)	48.37 (±2.43)	0.002	16.57 (±3.13)	–
CelA2-H288F	0.50 (±0.022)	8.95 (±1.62)	0.056	72.62 (±3.21)	H288F
CelA2-H288F-M1	1.52 (±0.046)	9.66 (±1.19)	0.157	220.60 (±6.71)	N273D/H288F/N468S

One Unit was defined as the amount of cellulase that catalyzes the conversion of 1 μmol of 4-MUC per minute. All reported values are the average of three measurements and deviations are calculated from the corresponding mean values. Values are normalized to protein content based on Bio-Rad chip analysis.
