# Supplementary material for: In vitro flow cytometry-based screening platform for cellulase engineering
Source: Sci Rep. 2016 May 17;6:26128. doi: 10.1038/srep26128 (PMC4869107; doi:10.1038/srep26128)
Supplement: Supplementary Information [file srep26128-s1.pdf]

Submitted to

Scientific Reports

Section

Article / Full manuscript

Supplementary data

In vitro flow cytometry-based screening platform for cellulase engineering

Georgette Körfer[b], Christian Pitzler[b], Ljubica Vojcic[b], Ronny Martinez[b], and Ulrich Schwaneberg[a,b]*

[a] DWI an der RWTH Aachen e.V, Forckenbeckstraße 50, 52056 Aachen, Germany
[b] RWTH Aachen University, Worringerweg 3, D-52074 Aachen, Germany

*Corresponding author: Prof. Dr. Ulrich Schwaneberg
RWTH Aachen University
Lehrstuhl für Biotechnologie
Gebäude 2. Sammelbau Biologie
Raum 4.139
Worringerweg 3
52074 Aachen
Tel.: +49 241 80 24170
Fax: +49 241 80 22578
E-mail: u.schwaneberg@biotec.rwth-aachen.de

Supplementary Data

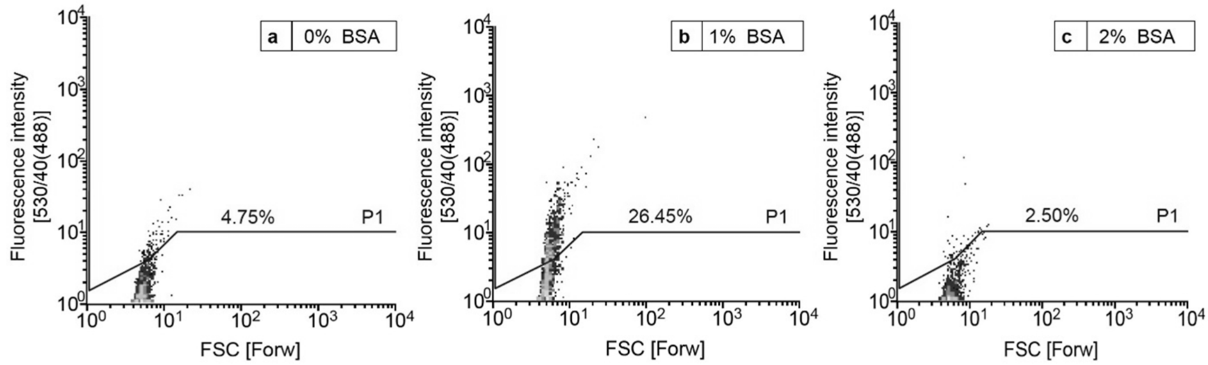

Figure S1: Flow cytometer analysis showing density plots obtained after cell-free production of cellulase in (w/o/w) emulsion containing varied BSA concentrations (4 h, 25°C). (**a**) (W/o/w) emulsion reaction sample incubated without BSA, (**b**) incubated with 1 % BSA, and (**c**) incubated with 2 % BSA. The forward scatter (FSC) on the x-axis represents the size of (w/o/w) emulsions. The y-axis represents the fluorescence intensity after 488 nm excitation with a fluorescence emission detection between 530±20 nm which is annotated by 530/40(488). Lines indicate gate P1 which was set to categorize events in highly fluorescent and potentially beneficial events (appearing above) and not beneficial events.

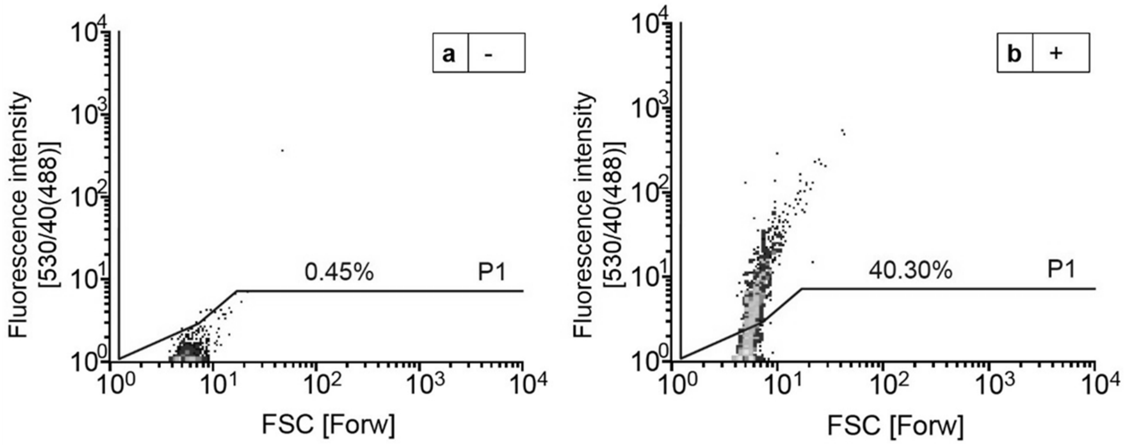

Figure S2: Flow cytometer analysis showing density plots obtained after cell-free production of cellulase in (w/o/w) emulsion after 4 h incubation at 25°C. (**a**) Negative control using CelA2-H288F-E580Q$_{inactive}$ for determination of background, (**b**) positive control using only CelA2-H288F$_{active}$. The forward scatter (FSC) on the x-axis represents the size of w/o/w emulsions. The y-axis represents the fluorescence intensity after 488 nm excitation with a fluorescence emission detection between 530±20 nm which is annotated by 530/40(488). Lines indicate gate P1 which was set to categorize events in highly fluorescent and potentially beneficial events (appearing above) and not beneficial events.

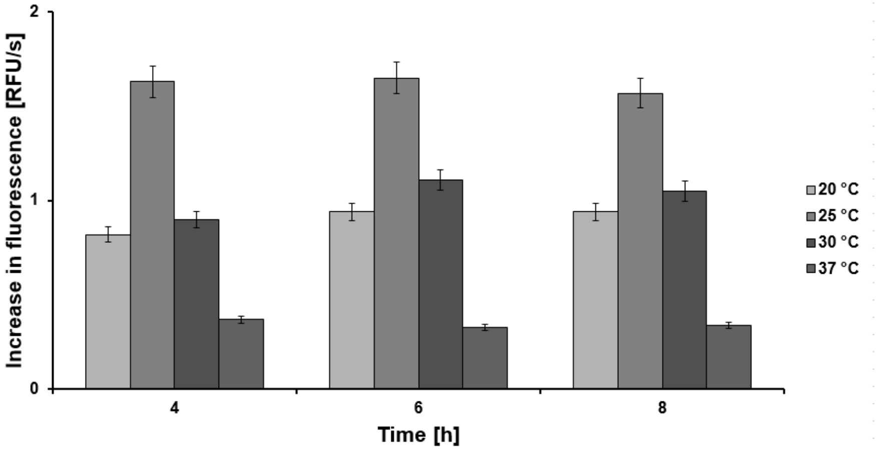

Figure S3: Variation of incubation temperature (20-37°C) and incubation time (4-8 h) for optimization of cell-free cellulase production. The time is shown on the x-axis and the activity is determined as increase in fluorescence in relative fluorescent units (RFU) per second, represented on the y-axis. Coefficient of variations was less than 5 %.

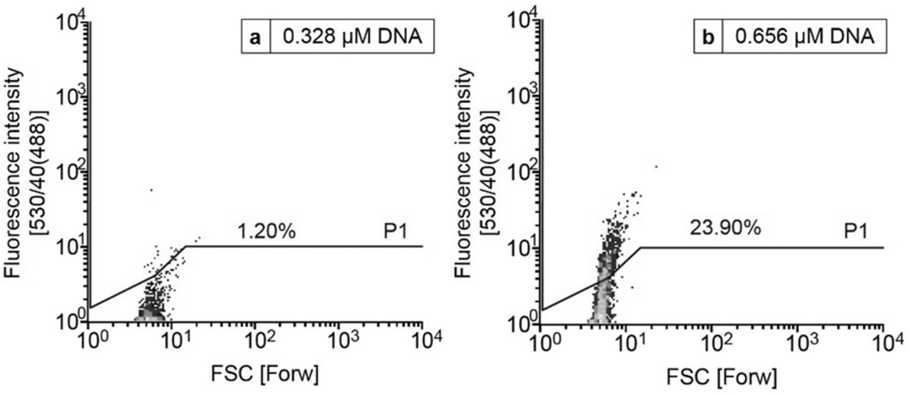

Figure S4: Flow cytometer analysis showing density plots obtained after cell-free production of cellulase in (w/o/w) emulsion with two amounts of linear DNA (**a**) 0.328 and (**b**) 0.656 µM DNA employing a model library with 30 % of active variants (ratio 3:7 of CelA2-H288F$_{\text{active}}$ versus CelA2-H288F-E580Q$_{\text{inactive}}$). The forward scatter (FSC) on the x-axis represents the size of (w/o/w) emulsions. The y-axis represents the fluorescence intensity after 488 nm excitation with a fluorescence emission detection between 530±20 nm which is annotated by 530/40(488). Lines indicate gate P1 which was set to categorize events in highly fluorescent and potentially beneficial events (appearing above) and not beneficial events.

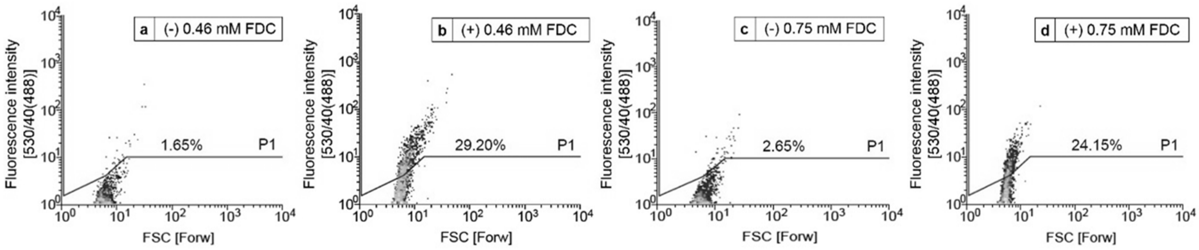

Figure S5: Flow cytometer analysis showing density plots obtained after cell-free production of cellulase in (w/o/w) emulsion with two substrate concentrations (0.46 and 0.75 mM FDC) in order to optimize the signal-to-noise-ratio. As negative control CelA2-H288F-E580Q$_{\text{inactive}}$ and as positive control CelA2-

H288F$_{active}$ were used to discriminate between background and cellulase activity. In detail, (**a**) negative control with 0.46 mM FDC, (**b**) positive control with 0.46 mM FDC, (**c**) negative control with 0.75 mM FDC, and (**d**) positive control with 0.75 mM FDC. The forward scatter (FSC) on the x-axis indicated the size of (w/o/w) emulsions. The y-axis represents the fluorescence intensity after 488 nm excitation with a fluorescence emission detection between 530±20 nm which is annotated by 530/40(488). Lines indicate gate P1 which was set to categorize events in highly fluorescent and potentially beneficial events (appearing above) and not beneficial events.

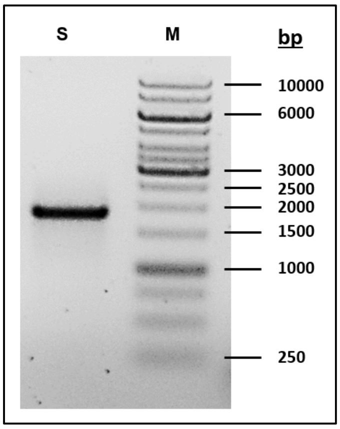

Figure S6: Results of a sorted InVitroFlow campaign**.** Agarose-gelelectrophoresis picture of recovered and amplified PCR products after DNA isolation from sorted (w/o/w) emulsions using NucleoSpin® Gel and PCR clean up kit (Macherey-Nagel). M represents the marker (GeneRuler^{TM} 1 kb DNA Ladder, Life Technologies GmbH, Darmstadt, Germany) used as reference. S represents the recovered amplified DNA (~1.8 kb) from the active emulsion fractions sorted with flow cytometer from an epPCR library (0.05 mM $MnCl_2$). The DNA was isolated with NucleoSpin® Gel and PCR clean up kit (Machery Nagel).

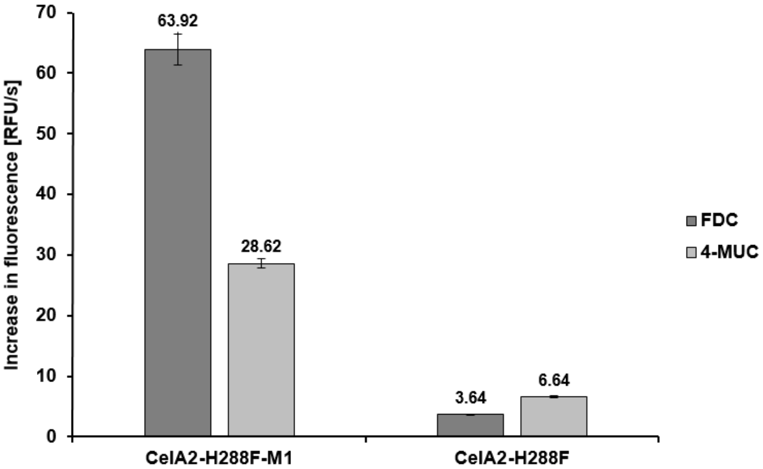

Figure S7: Results of CelA2-H288F-M1 and parent CelA2-H288F analysis with FDC and 4-MUC activity assay in MTP format using crude cell extracts. The x-axis describes the two different variants and the y-axis shows the increase in fluorescence in relative fluorescent units (RFU) per s. The reported values are the average of two measurements and the shown average deviations are calculated from the mean values. For the activity assay crude cell extract (40 µl) was mixed with FDC or 4-MUC substrate (0.05 mM; 20 µl) and potassium phosphate buffer (pH 7.2, 0.2 M; 40 µl).
